# Diastolic function imaging: a comparison of real-time phase contrast magnetic resonance (CMR) imaging with segmented phase contrast CMR and Doppler echocardiography

**DOI:** 10.1186/1532-429X-14-S1-W21

**Published:** 2012-02-01

**Authors:** Paaladinesh Thavendiranathan, Jacob A Bender, Jennifer Dickerson, Michael Pennell, Alice M Hinton, Subha V Raman, Orlando P Simonetti

**Affiliations:** 1 Cardiovascular Medicine, Cleveland Clinic Foundation, Cleveland, OH, USA; 2 Cardiovascular Medicine, The Ohio State University, Columbus, OH, USA

## Background

CMR measurement of mitral inflow velocities for the assessment of diastolic function is often infeasible in patients with dyspnea - patients who may benefit the most - due to their inability to breath-hold. Although real-time phase contrast (RT-PC) imaging may overcome this limitation, it has not been systematically evaluated. The objective of this study was to assess the accuracy of RT-PC for the measurement of mitral inflow velocities against segmented PC CMR and Doppler echocardiography.

## Methods

37 healthy volunteers (aged 28 ± 10 years, 20 males) had echo and CMR studies within a week. Early (E) and late (A) mitral inflow velocities were measured by echo, segmented, and RT-PC CMR (Figure). The E and A velocities were obtained by averaging data from 2 heart beats by RT-PC and 3 heart beats by echo. RT-PC parameters were: TR/TE = 14.0ms/2.3ms, water excitation flip angle=25○,10mm slice, 90 x128 matrix, EPI factor=15, TSENSE rate=3, and VENC=150cm/s. Shared velocity encoding was used to achieve an effective temporal resolution of 28ms, but true temporal resolution was 56ms. Retro-gated segmented PC acquisition parameters: TR/TE = 4.5/1.9ms, 10mm slice, 100 x 192 matrix, TSENSE rate=3, VENC=150cm/s, true temporal resolution 36ms. E and A velocities, and E/A ratios between RT-PC and segmented PC CMR or Doppler echocardiography were compared using paired t-tests. Agreement between the techniques was assessed using concordance correlation coefficients and Bland-Altman analysis.

**Figure 1:**
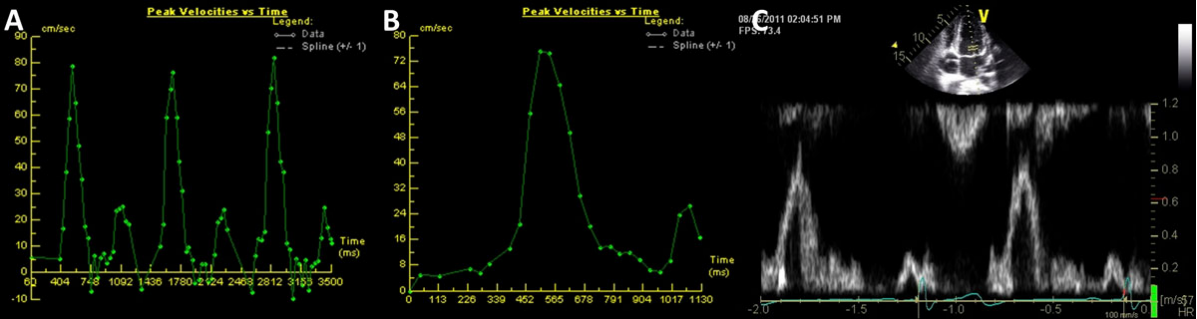
Mitral inflow velocities in one volunteer by all three techniques: (A) real-time phase contrast imaging (mean E, A, and E/A were 78cm/s, 25cm/s, and 3.1), (B) segmented phase contrast imaging (mean E, A, and E/A were 75cm/s, 27cm/s, and 2.8), and (C) Doppler echocardiography (mean E, A, and E/A were 77cm/s, 26cm/s, and 3.0).

## Results

Mean E velocities by echo, segmented, and RT-PC CMR were 75 ± 15 cm/s, 77 ± 12 cm/s, and 73 ± 12cm/s , respectively. The RT-PC measurements were not different from echo (p=0.3), but were less than segmented PC CMR (p=0.04). The A velocities (38 ± 12 cm/s, 38 ± 11 cm/s, 35 ± 12 cm/s, respectively) were not different between RT-PC CMR and echo or segmented CMR (p=0.3 for both). There was also no difference in the E/A ratios (2.2 ± 0.6, 2.2 ± 0.7, and 2.2 ± 0.9, respectively; p =0.6 for both). There was moderate concordance between RT-PC CMR and segmented CMR and Echo for E, A and E/A ratio (Table 1). Although, the bias in measurement between RT-PC CMR and echo or segmented CMR was small, the LOA was wide.

**Table 1:** 

	Concordance Correlation	Bland-Altman Analysis (Bias ± LOA)cm/s
RT-PC CMR vs Echo E	0.41	2.5 ± 26.4
RT-PC CMR vs Echo A	0.38	2.0 ± 24.7
RT-PC CMR vs. Echo E/A ratio	0.42	0.1 ± 1.8
RT-PC CMR vs Segmented E	0.56	3.9 ± 21.4
RT-PC CMR vs Segmented A	0.38	2.1 ± 24.4
RT-PC CMR vs Segmented E/A	0.42	-0.1 ± 1.6
Segmented PC CMR vs echo E	0.62	-1.4 ± 17.3
Segmented PC CMR vs echo A	0.72	-0.1 ± 13.9
Segmented PC CMR vs echo E/A	0.67	0.0 ± 1.2

CMR, cardiac magnetic resonance imaging; RT-PC, Real-time phase contrast CMR; echo, echocardiography; LOA, level of agreement; 2SD, 2 standard deviations

## Conclusions

We demonstrate for the first time the use of RT-PC imaging to measure mitral E and A velocities. There was modest agreement between RT-PC CMR and echo and segmented PC CMR. Further refinements of the RT-PC sequences are necessary; however, the use of RT-PC imaging provides an opportunity for wider application in patients who have difficulty with breath holding or arrhythmias.

## Funding

National Institute of Health (NIH, R01).

